# Editorial: Immune responses and immune mechanisms triggered by snake and scorpion venoms

**DOI:** 10.3389/fimmu.2022.988924

**Published:** 2022-09-13

**Authors:** Manuela B. Pucca, Denise V. Tambourgi, Wuelton M. Monteiro

**Affiliations:** ^1^ Medical School, Federal University of Roraima, Boa Vista, Roraima, Brazil; ^2^ Laboratory of Immunochemistry, Butantan Institute, São Paulo, Brazil; ^3^ Department of Research, Dr. Heitor Vieira Dourado Tropical Medicine Foundation, Manaus, Amazonas, Brazil; ^4^ School of Health Sciences, Amazonas State University, Manaus, Amazonas, Brazil

**Keywords:** snake-derived toxins, scorpion-derived toxins, immune system, immunomodulators, snakebite, scorpion sting, envenomation

Snake and scorpion venoms are composed of proteins and peptides, which makes them a cocktail of diverse bioactive molecules. These biomolecules are responsible for disturbing fundamental physiological systems of victims of envenomings. Nonetheless, researchers have turned life-threatening toxins into life-saving therapeutics, as well as important tools for investigating the mechanisms underlying the pathology of envenomings.

The immune system is one of the major physiological systems affected by snake and scorpion envenomings. The activation of the immune system is involved in the formation of edema, pain, neutrophil recruitment, activation of the complement system, and release of cytokines (e.g., IL-1β, IL-6, IL-10, and TNF-α) and other inflammatory mediators such as PGE2 and LTB4. Thus, the comprehension of the mechanisms underlying the modulating action of snake and scorpion venom-derived toxins in the immune system may help to understand the molecular basis of envenomings, as well as enable us to design novel molecules for immunomodulation for numerous diseases.

In this article collection, the authors show new data and reviews regarding the immune responses and immune mechanisms that are triggered by snake and scorpion venoms, besides presenting new approaches for drug discovery and diagnosis. At last, a pioneer review article regarding the *Bothops bilinetus* snake, two-lined forest pitviper is presented.

## Innate immunity

Although the complement system is recognized as an important defense mechanism of innate immunity, its over activation has been linked to contributing to the pathophisiology of snake and scorpion envenomings. Using an *ex vivo* model of human whole blood, Leonel et al. show that the venom of the snake *B. jararaca* promotes activation of the complement system, which significantly contributes to the inflammatory process. Moreover, the authors demonstrated that the control of several inflammatory parameters using inhibitors of the complement system indicates that complement inhibition may represent a potential therapeutic tool in *B. jararaca* envenomings. In addition, the role of cells from innate immunity during envenomings have also been investigated. By using mice selected for maximum (AIRmax) and minimum (AIRmin) inflammatory response, Kondo et al. analyzed the role of mast cells in the inflammatory process induced by the venom of *Bothrops jararaca*. The results show that mast cells are involved in pain, neutrophil migration, and in ROS production triggered by the snake venom in AIRmax mice, which are more susceptible to *B. jararaca* venom.

## Drug discovery

The standard treatment for snakebite envenomings is the administration of antivenom, which is produced through hyperimmunization of horses with snake toxins. Due to cold-chain requirements, efficacy and safety limitations of the currently available antivenoms, there has been rapidly growing interest in the prospection and pre-clinical characterization of molecules that can complement the treatment with antivenom. Chowdhury et al. demonstrated that varespladib was able to neutralize *Naja ashei*, *N. katiensis*, and *N. nubiae* venom-derived toxins. In addition, the authors showed that prinomastat inhibited the FXa-inhibiting PLA2 toxins of all the African spitting cobras at the same concentration at which it has been shown to inhibit metalloproteases and, thus, was comparably effective in its cross-reactivity. Adrião et al. discussed the potential of biodiversity-derived molecules represented by plant extracts. In this paper, the authors present 117 secondary metabolites which belong to different chemical classes (e.g., *alkaloids, benzenoids, flavonoids, isoflavonoids, polyketides, terpenes, saponins, and others*), that are able to inhibit toxins, thus demonstrating promising sources of novel medicines to treat or minimize snakebite envenomings.

## Diagnosis

Due the complexity of venom compositions, venom diagnostic methods for genus or species-specific identification has been considered a huge challenge. Long et al. developed a strategy to select and prepare genus-specific snake venom antibodies, which allows rapid and efficient clinical diagnosis of snakebites. Based on this strategy, the authors successfully developed diagnostic antibodies against the *Trimeresurus* and *Protobothrops* snake genera, which are responsible for hundreds of thousands of snakebites each year in China.

## *B. bilineatus* pioneer review article

Bernarde et al. performed an extensive literature review on the two-striped forest-pitviper (*Bothrops bilineatus*), a medically important species in South America that is distributed in the Amazon and the Atlantic Forest. The major components of *B. bilineatus* venom are snake venom metalloproteinases (SVMPs), with the predominance of the PIII-class, snake venom serine proteinases (SVSP), C-type lectin-like proteins (CTL), phospholipases A2 (PLA2), cysteine-rich secretory proteins (CRISP), L-amino acid oxidases (LAAO), and bradykinin-potentiating-like peptides (BPPs). These toxins interact with the patient’s immune system and hemostasis, which leads to local inflammatory reaction, local tissue damage, and dysfunctions in coagulation. Strikingly, none of the antivenom-manufacturing laboratories in South America include *B. bilineatus* venom as an antigen, but preliminary studies show that the *Bothrops* antivenom available in Brazil was able to recognize components of *B. bilineatus* venom.

## Concluding remarks

Snake and scorpion venoms are complex mixtures of protein components, peptides, and other molecules that are capable of stimulating the immune system in different ways. However, compared to infectious and chronic inflammatory diseases, studies of the immune mechanisms triggered by snakebites and scorpion stings have historically been studied with low priority. Nonetheless, it is increasingly clear that the understanding of immune mechanisms triggered by snake and scorpion venoms is essential in order to propose new innovative forms of treatment, including more effective and safe antivenoms, as weel as for the discovery of diagnostic and prognostic biomarkers. This Research Topic intends to contribute to the expansion of knowledge on this relevant and neglected issue.

## Author contributions

All the listed authors have made a substantial, direct and intellectual contribution to the work, and approved it for publication.

## Conflict of interest

The authors declare that the research was conducted in the absence of any commercial or financial relationships that could be construed as a potential conflict of interest.

## Publisher’s note

All claims expressed in this article are solely those of the authors and do not necessarily represent those of their affiliated organizations, or those of the publisher, the editors and the reviewers. Any product that may be evaluated in this article, or claim that may be made by its manufacturer, is not guaranteed or endorsed by the publisher.

